# Polycystic kidney disease 2-like 1 channel contributes to the bitter aftertaste perception of quinine

**DOI:** 10.1038/s41598-023-31322-3

**Published:** 2023-03-15

**Authors:** Takahiro Shimizu, Takuto Fujii, Keisuke Hanita, Ryo Shinozaki, Yusaku Takamura, Yoshiro Suzuki, Teppei Kageyama, Mizuki Kato, Hisao Nishijo, Makoto Tominaga, Hideki Sakai

**Affiliations:** 1 Department of Pharmaceutical Physiology, Faculty of Pharmaceutical Sciences, University of Toyama, Toyama, 930-0194 Japan; 2 Department of System Emotional Science, Faculty of Medicine, University of Toyama, Toyama, 930-0194 Japan; 3 Division of Cell Signaling, National Institute for Physiological Sciences, National Institutes of Natural Sciences (NIPS), Okazaki, 444-8787 Japan

**Keywords:** Cell biology, Physiology

## Abstract

Bitterness is an important physiological function in the defense responses to avoid toxic foods. The taste receptor 2 family is well known to mediate bitter taste perception in Type II taste cells. Here, we report that the polycystic kidney disease 2-like 1 (PKD2L1) channel is a novel sensor for the bitter aftertaste in Type III taste cells. The PKD2L1 channel showed rebound activation after the washout of quinine, a bitter tastant, in electrophysiological whole-cell recordings of the PKD2L1-expressing HEK293T cells and Ca^2+^-imaging analysis of Type III taste cells isolated from wild-type PKD2L1 mice. In the short-term two-bottle preference and lick tests in vivo, the wild-type mice avoided normal water while the PKD2L1-knockout mice preferred normal water after they ingested the quinine-containing water. These results may explain the new mechanism of the quinine-triggered bitter aftertaste perception in Type III taste cells.

## Introduction

Five basic tastes (sweet, umami, sour, salty, and bitter) are recognized by taste cells in the taste buds of the tongue. Taste cells are divided into Types I-IV, each of which has a different function^1^. Type I cells function as glial-like cells for the formation of taste buds. Type II cells sense sweet, umami, bitter, and salty tastes. Type III cells respond to a sour taste. Type IV cells are progenitor cells for Type I-III cells.

Bitterness is essential for a biological defense response to avoid toxic foods. The taste receptor 2 (T2R) family, a subfamily of G protein-coupled receptors, expresses in Type II taste cells and contributes to bitter taste perception^2–6^. In humans, there are 25 isoforms among the T2R family^7^, which recognizes various structurally diverse bitter compounds. However, the contribution of ion channels to bitter taste perception is unknown.

In the taste buds, the H^+^-selective ion channel, otoperin (OTOP1), and the intracellular pH-sensitive K^+^ channel, Kir.2.1, are involved in the sour transduction of Type III taste cells. The amiloride-sensitive epithelial Na^+^ channel (ENaC) is involved in the salty (sodium taste) transduction of Type II taste cells. The action potential–activated calcium homeostasis modulator 1/3 (CALHM1/3) ion channel is a key molecule in the channel synapse of Type II taste cells^8^.

Polycystic kidney disease 2-like 1 (PKD2L1), a member of the transient receptor potential (TRP) family^9^, is expressed in Type III taste cells of the circumvallate papillae in the mouse tongue^10^ and forms a heterocomplex with PKD1-like 3 (PKD1L3)^11^. The heterocomplex functions as a Ca^2+^-permeable non-selective cation channel and responds to sour taste^11–14^. On the contrary, PKD1L3 knockout mice show normal acid taste receptivity^15,16^. Besides, PKD2L1-deficient mice retain the sour response^16^. Therefore, the physiological roles of PKD2L1 in taste perception remain unknown.

We previously demonstrated that the PKD2L1 channel is sensitive to alkalization and heating in the human embryonic kidney (HEK) 293 T cells exogenously expressing PKD2L1^17–19^. Interestingly, the channel was activated after the removal of the alkaline stimuli, while the alkalization itself inactivated the channel.

Here, we found that the PKD2L1 channel is involved in sensing bitterness. Interestingly, the PKD2L1 channel contributes to sensing the quinine-induced bitter aftertaste.

## Results

### Quinine, a bitter compound, triggers a rebound activation of PKD2L1 channels

**Figure 1:**
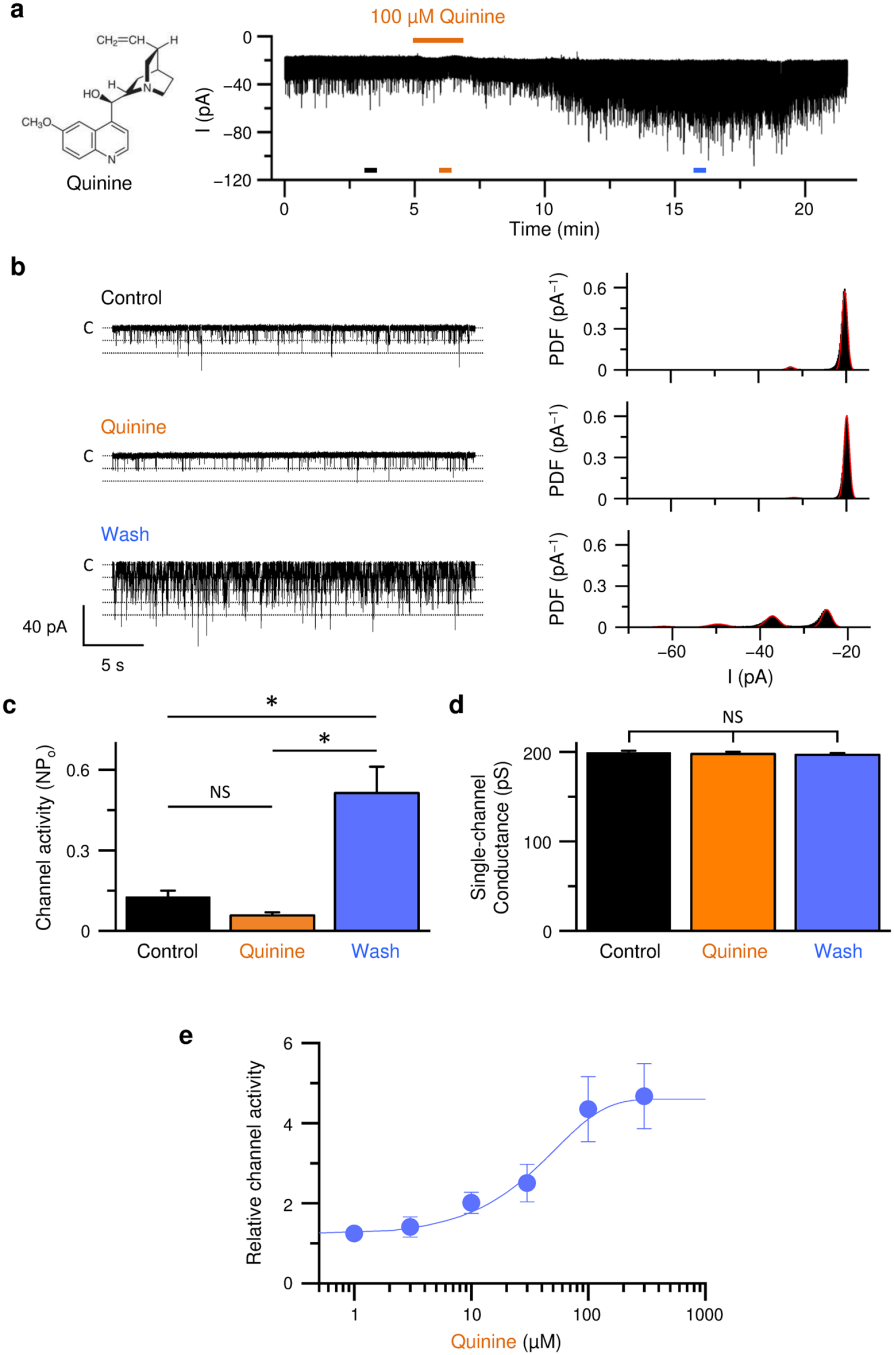
Quinine induced off-response of PKD2L1 channel. (**a**) Whole-cell PKD2L1 current recorded at − 60 mV in HEK293T cells exogenously expressing PKD2L1. Quinine (100 µM) was added as indicated by an upper *orange line*. (**b**) Time-expanded traces from the *horizontal bars* (*black*, *orange,* and *blue*) in (**a**). The traces before (Control, *black*), during (Quinine, *orange*), and after (Wash, *blue*) application of quinine are shown. *Dotted lines* indicate closed (C) and open levels. The corresponding all-points amplitude histograms are shown on the right. PDF stands for probability density function. Gaussian fittings are shown in *red lines*. (**c**, **d**) The channel activity (NPo) (**c**) and the single-channel conductance (**d**) before (Control), during (Quinine), and after (Wash) application of quinine. The data are averaged from 20 experiments. *P* values are determined by one-way ANOVA with Tukey’s post-hoc analysis (F = 17.59 in **c** and F = 0.24 in **d**). *, *P* < 0.05. NS, *P* > 0.05. (**e**) Concentration dependence of quinine on the off-response of the PKD2L1 channel activities at − 60 mV. The data are averaged from 6 to 18 experiments.

Quinine is a cinchona alkaloid exhibiting an antimalarial activity, which has been used as a standard bitter tastant in taste perception research^20^. In the present study, we investigated whether a bitterant, quinine, affects the constitutive PKD2L1 currents recorded in whole-cell patch-clamp recordings^17–19^. As shown in Fig. 1a, the inward PKD2L1 currents at − 60 mV were rather decreased during the application of quinine at 100 µM. Interestingly, the washout of quinine induced the long-lasting activation of PKD2L1 channels. Single-channel analysis revealed that the rebound activation of PKD2L1 channels is due to an increase in the channel activity (NPo) but not in the single-channel conductance (Fig. 1b–d). The channel activities after the removal of quinine (off-response) were increased in a concentration-dependent manner (Fig. 1e). The half-maximal effective concentration (EC_50_) was 33 µM (n = 6–18).

**Figure 2:**
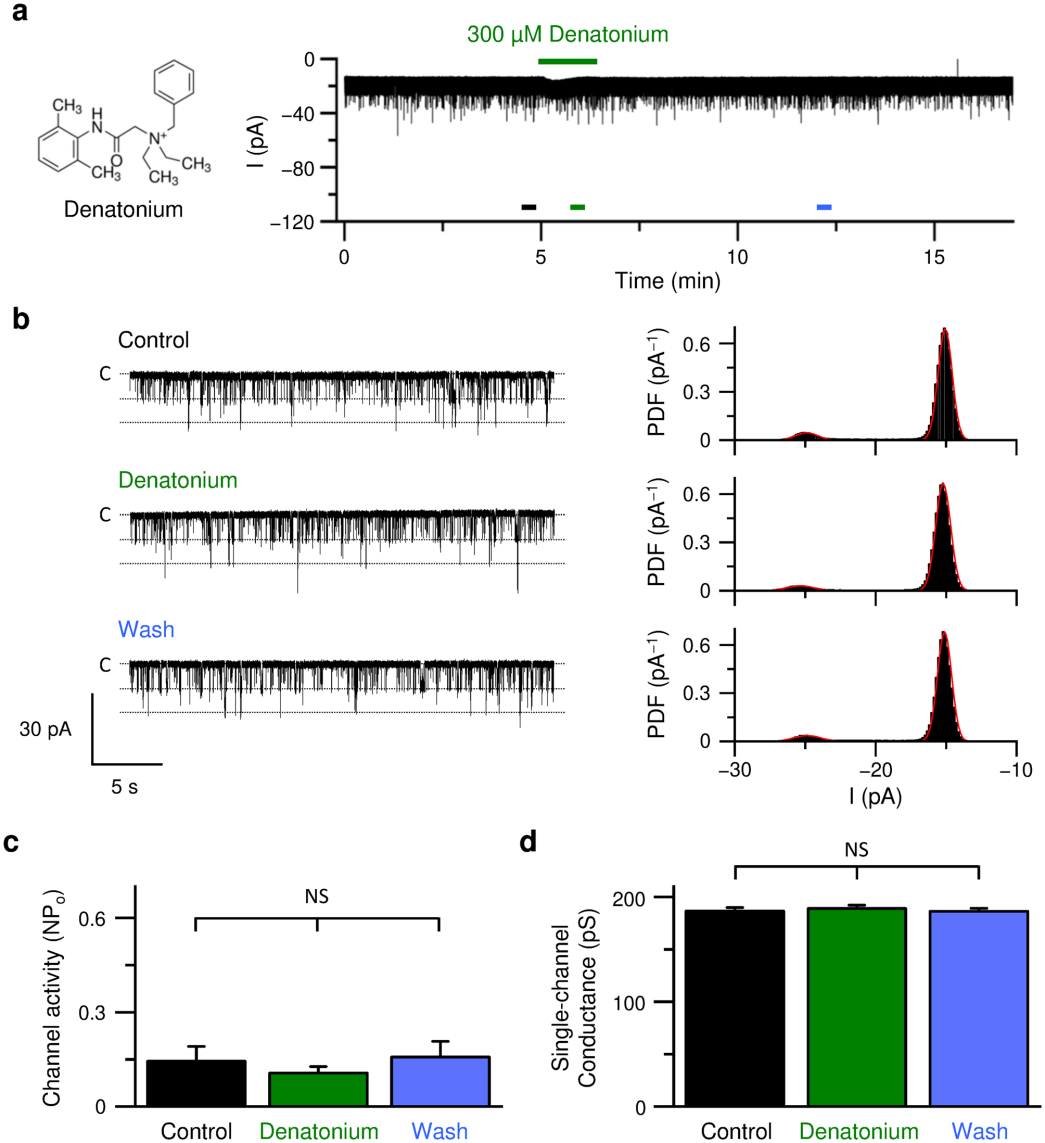
Denatonium had no effect on the PKD2L1 activity. (**a**) Whole-cell PKD2L1 current recorded at − 60 mV in HEK293T cells exogenously expressing PKD2L1. Denatonium (300 µM) was added as indicated by an upper *green line*. (**b**) Time-expanded traces from the *horizontal bars* (*black*, *green,* and *blue*) in (**a**). The traces before (Control, *black*), during (Denatonium, *green*), and after (Wash, *blue*) application of denatonium are shown. *Dotted lines* indicate closed (C) and open levels. The corresponding all-points amplitude histograms are shown on the right. PDF stands for probability density function. Gaussian fittings are shown in *red lines*. (**c**, **d**) The channel activity (NPo) (**c**) and the single-channel conductance (**d**) before (Control), during (Denatonium), and after (Wash) application of denatonium. The data are averaged from nine experiments. *P* values are determined by one-way ANOVA with Tukey’s post-hoc analysis (F = 0.41 in **c** and F = 0.23 in **d**). NS, *P* > 0.05.

In contrast, another bitter compound, denatonium, at 300 µM showed no off-response of the PKD2L1 channel after the removal of denatonium (Fig. 2).

### Quinine increases intracellular free Ca^2+^ concentration ([Ca^2+^]_i_) via PKD2L1 channels in mouse Type III taste cells

PKD2L1 is endogenously expressed in mouse Type III taste cells^10^. Here, we investigated whether quinine and denatonium affect [Ca^2+^]_i_ through the activation of PKD2L1. Since Type III taste cells express voltage-dependent Ca^2+^ channels^1^, we could easily identify the cells. In fact, they show the depolarization-induced elevation of [Ca^2+^]_i_ upon exposure to the high-K^+^ solution.

**Figure 3:**
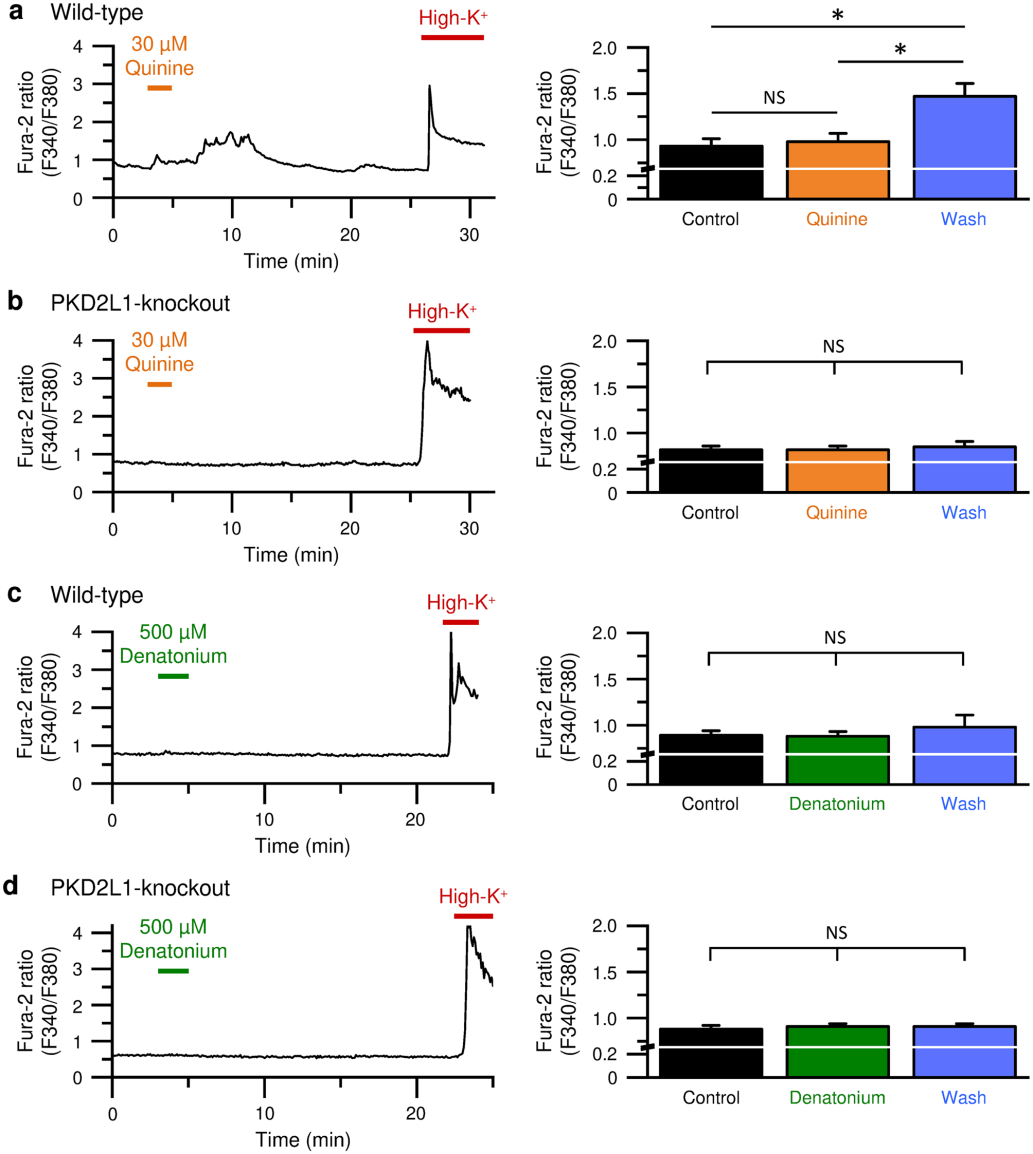
Quinine, but not denatonium, triggered off-response of the PKD2L1 channel in isolated Type III taste cells from mice. (**a**–**d**) Representative traces of intracellular free Ca^2+^ concentration measured as Fura-2 ratio (Left) and the averaged Fura-2 ratio (Right) before (Control), upon (Quinine or Denatonium), and after (Wash) an application of 30 µM quinine (**a**, **b**) or 500 µM denatonium (**c**, **d**) in Type III taste cells isolated from the wild-type (**a**, **c**) or the PKD2L1-knockout (**b**, **d**) mice. The data are averaged from ten experiments. *P* values are determined by one-way ANOVA with Tukey’s post-hoc analysis (F = 8.08 in **a**, F = 0.11 in **b**, F = 0.36 in **c**, and F = 0.17 in **d**). *, *P* < 0.05. NS, *P* > 0.05.

In Type III taste cells isolated from the wild-type mice, quinine at 30 µM did not significantly affect [Ca^2+^]_i_ during its application (Fig. 3a), However, [Ca^2+^]_i_ was elevated after the removal of quinine. To clarify whether the Ca^2+^-permeable PKD2L1 channel contributes to the quinine-induced Ca^2+^ response, we prepared isolated Type III taste cells from the PKD2L1-knockout mice. In contrast to the wild-type cells, the PKD2L1-knockout cells had no response to quinine even after its removal (Fig. 3b).

Consistent with the results in electrophysiological experiments (Fig. 2), denatonium at 500 µM had no effect on [Ca^2+^]_i_ during and after its application in the wild-type (Fig. 3c) and the PKD2L1-knockout cells (Fig. 3d).

PKD2L1 deficiency induces the loss of β2-adrenergic receptor (β2AR) on neuronal primary cilium in the mouse brain and reduces cAMP signals^21^. Here, we compared the expression of the β2AR in the circumvallate papillae isolated from the wild-type and the PKD2L1-knockout mice. The quantitative polymerase chain reaction (qPCR) analysis showed that the mRNA level of the β2AR normalized to GAPDH in the wild-type mice (1.56 ± 0.14, n = 3) was similar to that in the PKD2L1-knockout mice (1.86 ± 0.10, n = 3, *P* > 0.05). In addition, we examined intracellular cAMP levels in the circumvallate papillae isolated from the wild-type and the PKD2L1-knockout mice. The cAMP levels after the treatment with 30 µM quinine were comparable in the wild-type (1.29 ± 0.02 pmol/ml, n = 3) and the PKD2L1-knockout mice (1.44 ± 0.14 pmol/ml, n = 3, *P* > 0.05).

### Avoidance of quinine-containing water by mice in vivo

**Figure 4:**
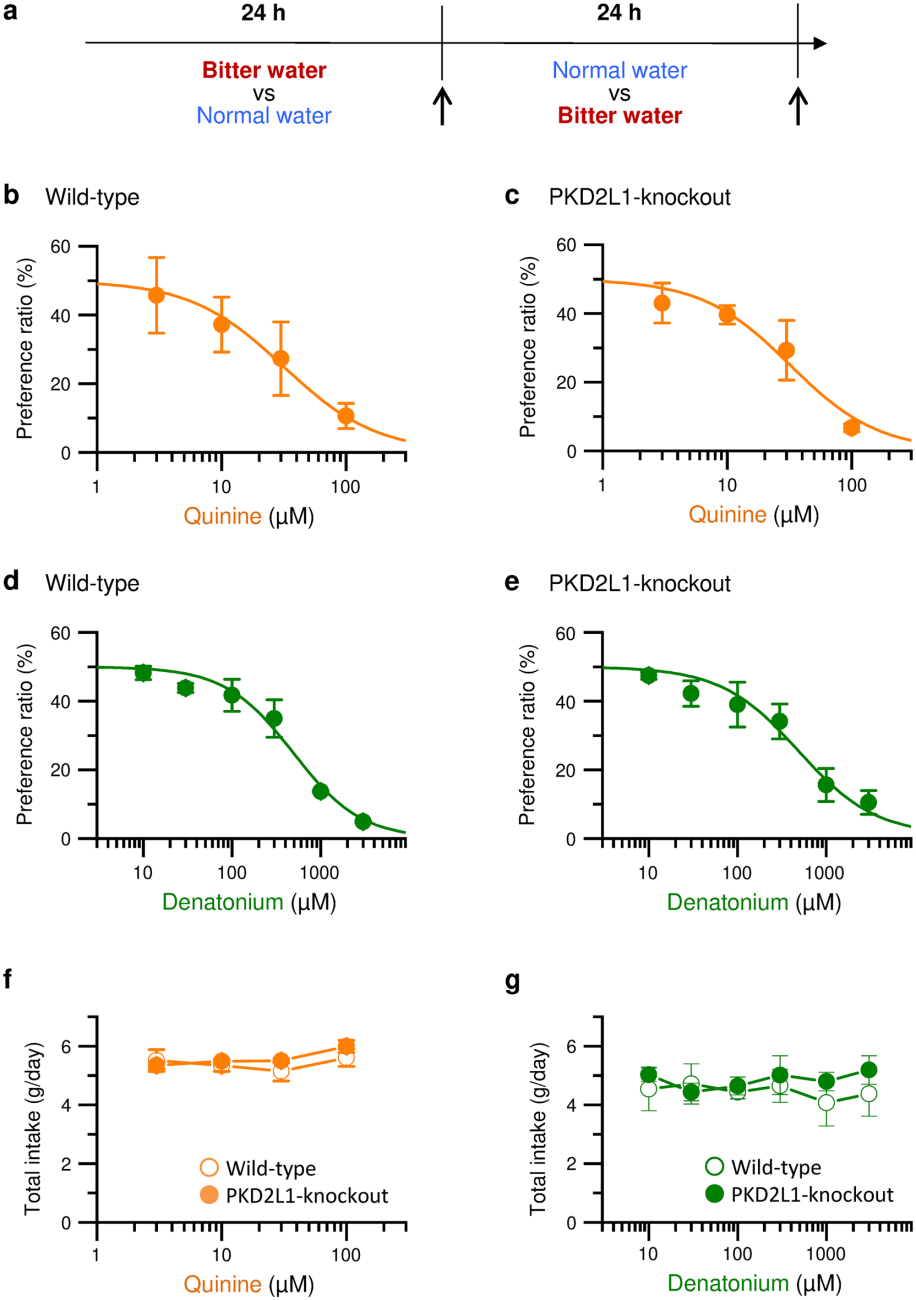
Intakes of quinine- or denatonium-containing water in long-term two-bottle preference tests in mice. (**a**) Timeline of experimental protocols. *Arrows* indicate the timing that two bottles were replaced and the intake was measured. (**b**–**e**) Behavioral responses to 3–100 µM quinine (**b**, **c**) or 10–3000 µM denatonium (**d**, **e**) in the wild-type (**b**, **d**) or the PKD2L1-knockout (**c**, **e**) mice. Two bottles of bitterant-containing water and normal water were given for 48 h. The preference ratio of 50% means that the intake of bitterant-containing water is the same as normal water. (**f**, **g**) Total amount of intakes of normal water and quinine- (**f**) or denatonium-containing water (**g**) per day by the wild-type and the PKD2L1-knockout mice. *P* values are determined by unpaired two-tailed Student’s t-test. The data are averaged from three experiments.

In long-term two-bottle preference tests, the wild-type and the PKD2L1-knockout mice were given two different bottles (one is normal water and another is quinine (3–100 µM)- or denatonium (10–3000 µM)-containing water). The amount of intake for 48 h in each bottle was measured (Fig. 4a). As shown in Fig. 4b, the wild-type mice avoided ingesting quinine-containing water in a concentration-dependent manner. The half-maximal effective concentration (EC_50_) was 31 µM. The PKD2L1-knockout mice also avoided ingesting quinine-containing water with the EC_50_ of 32 µM (Fig. 4c). Similarly, both the wild-type and the PKD2L1-knockout mice avoided ingesting denatonium-containing water in a concentration-dependent manner (Fig. 4d and e). The EC_50_ in the wild-type mice (487 µM) was comparable to that in the PKD2L1-knockout mice (485 µM). In these experiments, the total amount of intakes of normal water and quinine- or denatonium-containing water per day by the wild-type mice were not significantly different from those by the PKD2L1-knockout mice (Fig. 4f and g). These results suggest that the PKD2L1-knockout mice have a normal mechanism for recognizing the bitterness of quinine as well as denatonium.

### PKD2L1 contributes to the aftertaste perception of quinine in vivo

**Figure 5:**
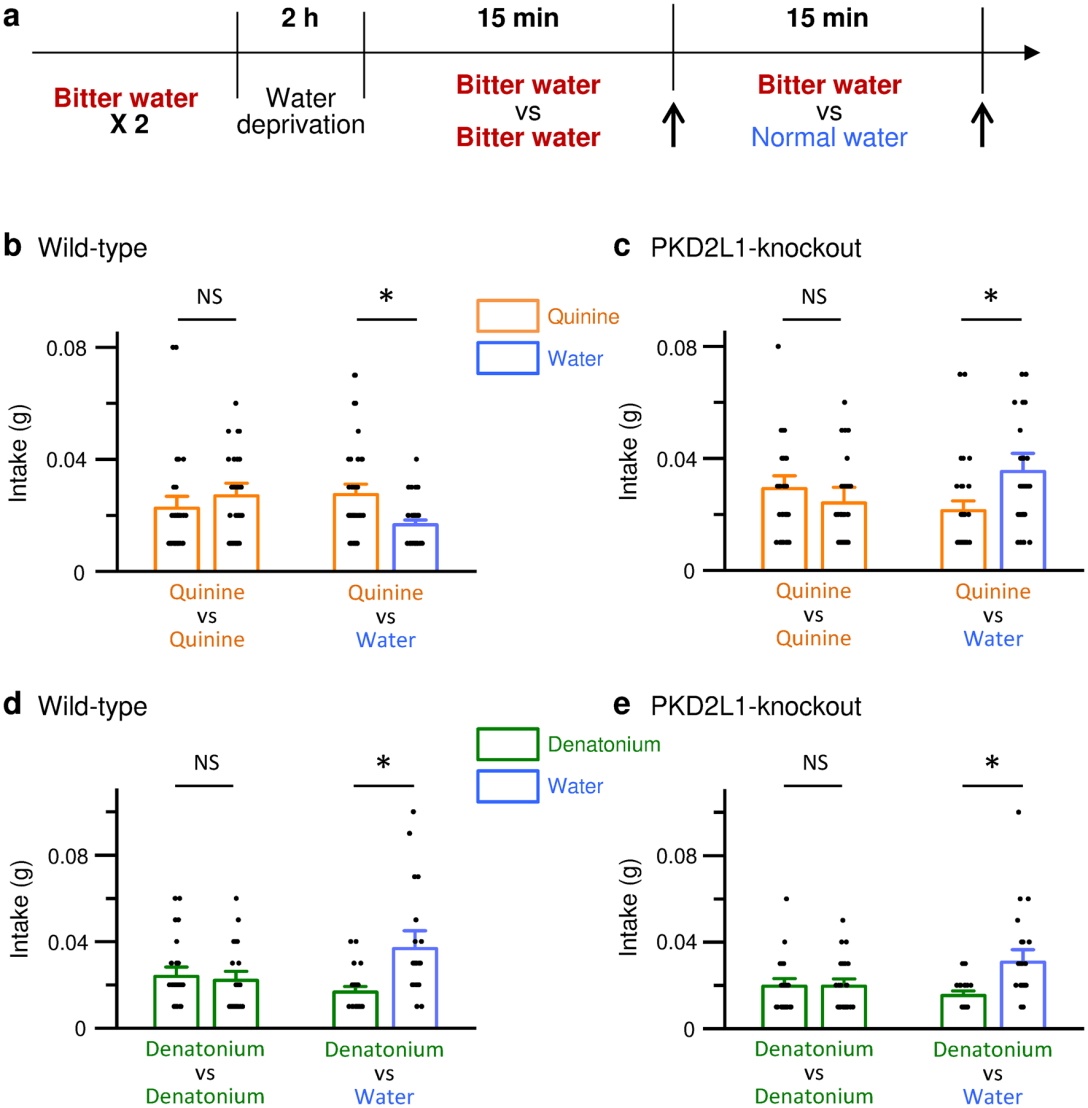
Intakes of quinine- or denatonium-containing water in short-term two-bottle preference tests in mice. (**a**) Timeline of experimental protocols. *Arrows* indicate the timing that two bottles were replaced and the intake was measured. (**b**–**e**) Mean intakes of 30 µM quinine- (**b**, **c**) or 500 µM denatonium- (**d**, **e**) containing water and normal water in the wild-type (**b**, **d**) or the PKD2L1-knockout (**c**, **e**) mice. Two bottles of bitterant-containing water were initially presented for 15 min and then bitterant-containing water and normal water were given for another 15 min. The data are averaged from 26 to 47 experiments. *P* values are determined by Wilcoxon signed-rank test (n = 47 in **b**, n = 39 in **c**, n = 28 in **d**, and n = 26 in **e**). *, *P* < 0.05. NS, *P* > 0.05.

Do the PKD2L1 channels contribute to the aftertaste of quinine in vivo? We conducted short-term two-bottle preference tests. In the experiments, two bottles of quinine-containing water were initially presented to mice for 15 min. Then, mice were given two different bottles (one is normal water and another is quinine-containing water) for 15 min. The amount of intake for 15 min in each bottle was measured (Fig. 5a). As shown in Fig. 5b, the wild-type mice that comparably drank the quinine-containing water in two bottles for the first 15 min avoided ingesting normal water for another 15 min. In contrast to the wild-type mice, the PKD2L1-knockout mice preferred to ingest normal water for another 15 min (Fig. 5c). On the other hand, both the wild-type and the PKD2L1-knockout mice that comparably drank the denatonium-containing water in two bottles for the first 15 min preferred to ingest normal water for another 15 min (Fig. 5d and e). These results suggest that the PKD2L1 channel contributes to the aftertaste perception of quinine but not denatonium.

**Figure 6:**
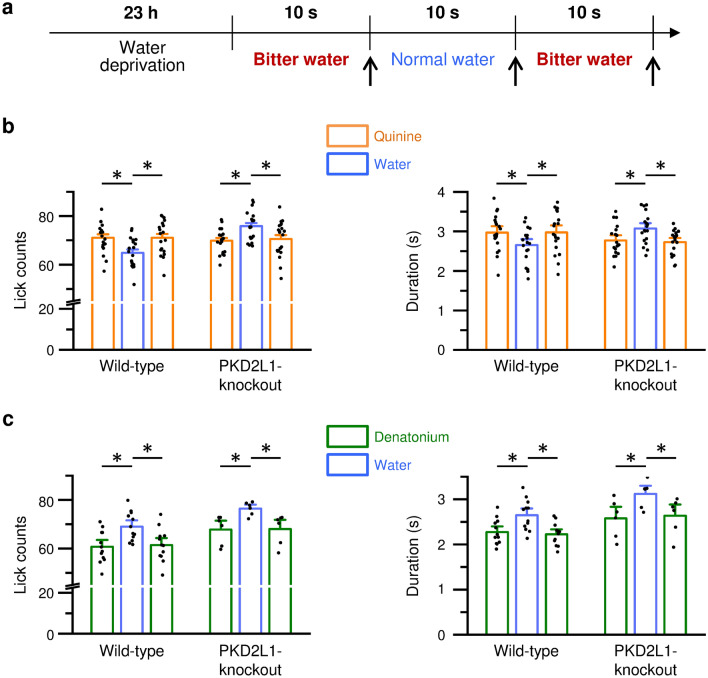
Lick behaviors to quinine or denatonium in mice. (**a**) Timeline of experimental protocols. *Arrows* indicate the time that a bottle was replaced. The counts and duration of licks were measured for each 10 s. (**b**, **c**) Lick counts (Left) and duration (Right) during the alternate presentation of 30 µM quinine-containing water and normal water (**b**) or 500 µM denatonium-containing water and normal water (**c**) in the wild-type and the PKD2L1-knockout mice. The data are averaged from 6 to 21 experiments. *P* values are determined by paired two-tailed Student’s t-test (n = 19/WT and n = 21/KO in **b**, n = 12/WT, and n = 6/KO in **c**). *, *P* < 0.05.

We next performed lick tests in the wild-type and the PKD2L1-knockout mice. In the tests, mice were alternately given a bottle of quinine-containing water or normal water for 10 s. Then, we measured the counts and duration of licks every 10 s (Fig. 6a). In the wild-type mice, the counts and duration of normal water were significantly reduced compared with those of quinine-containing water (Fig. 6b, left columns). In the PKD2L1-knockout mice, the counts and duration of normal water were significantly increased compared with those of quinine-containing water (Fig. 6b, right columns). On the other hand, the counts and duration of normal water were significantly greater than those of denatonium-containing water in both the wild-type and the PKD2L1-knockout mice (Fig. 6c). Consistent with Fig. 5, these results suggest that quinine but not denatonium triggers the aftertaste via the PKD2L1 channels.

## Discussion

**Figure 7:**
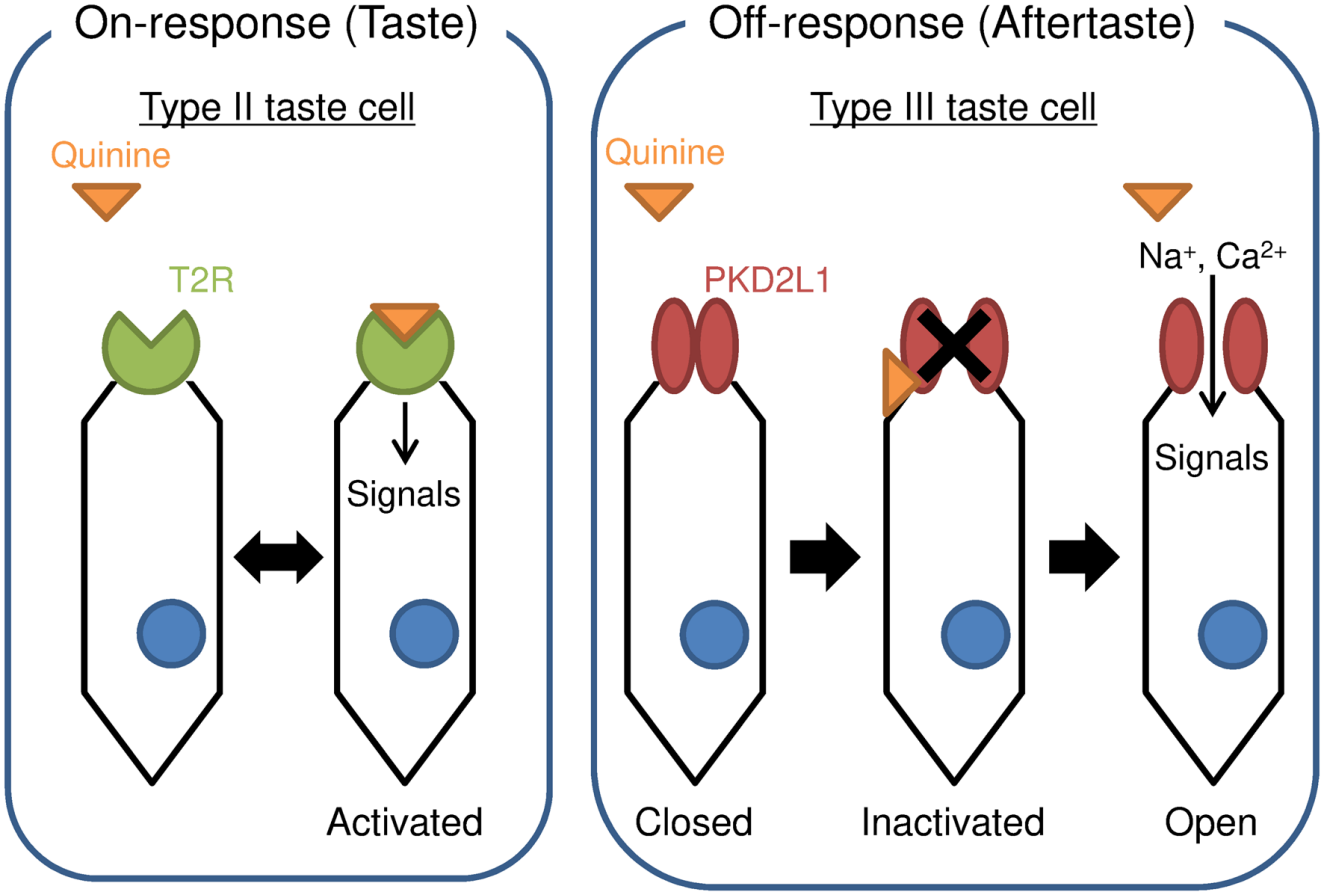
Proposed models of on- and off-response triggered by quinine. In Type II taste cells, quinine binds with the taste receptor 2 (T2R), a G-protein-coupled receptor, which triggers intracellular signals for bitter taste perception. On the other hand, quinine inactivated the PKD2L1 channel in Type III taste cells., The off-response of the PKD2L1 channel after the removal of quinine causes depolarization and elevation of [Ca^2+^]_i_, leading to a bitter aftertaste.

Normally, when we ingest bitter ingredients, we perceive two types of bitter taste: a taste in the presence of the compound and an aftertaste that persists after the removal of the compound. Previous reports showed that taste receptor 2 (T2R), a G-protein-coupled receptor, contributes to bitter taste reception in Type II taste cells^3,5,6^. In humans, there are 25 different T2R isoforms^7^, suggesting the diversity of bitter taste perception. The activation of T2R depends on the binding of bitter compounds, which causes an on-response to bitter taste (Fig. 7, left). On the other hand, the aftertaste is characterized by the intensity of stimulus lingering after the removal of tastants^22^. So far, the mechanism for recognizing the aftertaste has been poorly understood. We here propose a novel concept in which the PKD2L1 channel in Type III taste cells contributes to bitter aftertaste perception.

In the present study, the cells expressing PKD2L1 exhibited a rebound activation after the removal of quinine. The off-response to quinine was dependent on the PKD2L1 expression. Our results suggest that the delayed activation of the PKD2L1 channel by quinine could be implicated in the bitter aftertaste. We have previously found that the PKD2L1 channels show a rebound activation after the removal of alkaline stimuli and that the activation is mediated by the “inactivated” state of the channels^17^. We propose that quinine changes the states of the PKD2L1 channel from “closed” to “open” via the “inactivated” state (Fig. 7, right). In the “inactivated” state, the open channel pore is somehow plugged, thereby the PKD2L1 currents tend to decrease during an application of quinine. After washing out of quinine, the inactivated PKD2L1 channel becomes open, causing the rebound activation. Thus, the state transition of the PKD2L1 channel from “inactivated” to “open” may produce the off-response (Fig. 7, right).

The PKD2L1 channel exhibits voltage-dependent or Ca^2+^-dependent inactivation. In our previous study, the outer pore loop region of the PKD2L1 channel is demonstrated to be essential for its voltage-dependent inactivation^19^. On the other hand, the Ca^2+^-dependent inactivation is reported to be due to Ca^2+^-binding in the selectivity filter of the PKD2L1 channel^23^. At present, we do not know how quinine triggers the inactivation of the PKD2L1 channel. Further studies are required to clarify its inactivation mechanism.

Since the PKD2L1 is a Ca^2+^-permeable non-selective cation channel^24^, the rebound activation of the PKD2L1 channel by quinine initiates Ca^2+^ signaling in the Type III taste cells. Thereby synaptic vesicles are exocytosed from the Type III taste cells and the released neurotransmitters regulate the gustatory nerve activity. Previously, PKD2L1 deficiency was reported to decrease the intracellular cAMP concentration via the loss of the β2AR on neuronal primary cilium in the mouse brain^21^. In the present study, the expression level of the β2AR in the circumvallate papillae was not changed by the PKD2L1 knockout. Besides, the intracellular cAMP levels after the application of quinine in the wild-type circumvallate papillae were not significantly different from that in the PKD2L1-knockout circumvallate papillae. These results suggest that the β2AR-elicited cAMP pathway is not involved in the aftertaste perception of quinine,

The long-term two-bottle preference tests for 48 h demonstrated that the wild-type and the PKD2L1-knockout mice similarly avoided quinine-containing water. These results suggest that the on-response induced by the binding of quinine with T2Rs is predominant in the long-term experiments. Quinine and denatonium are promiscuous agonists of T2R^25^. Quinine and denatonium activate nine T2Rs (T2R4, 7, 10, 14, 31, 39, 40, 43, and 46) and eight T2Rs (T2R4, 8, 10, 13, 30, 39, 43, and 46), respectively. These T2Rs may contribute to the on-response of these bitter tastants. On the other hand, in the short-term two-bottle preference tests and the lick tests, the wild-type mice preferred quinine-containing water, while the PKD2L1-knockout mice preferred normal water. These results suggest that normal water intake after drinking quinine-containing water could induce the off-response of the PKD2L1 channel and enhance the bitter aftertaste of quinine in the wild-type mice.

In the present study, quinine and denatonium showed different effects on the off-response of the PKD2L1 channel, suggesting that quinine specifically interacts with the PKD2L1 channel. Quinine and denatonium are tertiary and quaternary amines, respectively (Figs. 1a and 2a), and their structures and properties are markedly different, which may account for the differences in their interactions with the PKD2L1 channel. Especially, the logarithm of the octanol–water partition coefficient (log P) estimated by the algorism developed at Molinspilation (milogP) of quinine and denatonium were 3.06 and − 0.62, respectively, indicating that quinine is more hydrophobic than denatonium. However, previous patch-clamp experiments using bullfrog taste receptor cells showed that both quinine and denatonium on the extracellular side directly activate the cation channels, but these tastants on the intracellular side do not affect the channels^26^. These results suggest that quinine and denatonium do not pass through the plasma membrane. Therefore, we propose that quinine may bind to the PKD2L1 channel at the extracellular vicinity of the plasma membrane, resulting in the delayed washout of quinine (Fig. 7, right). Further studies are required to understand the different reactivity of quinine and denatonium on the PKD2L1 channel.

The bitter perception is essential to avoid toxic substances as one of the biological defense reactions. Especially, the bitter taste of the toxic substances often long lasts. The persistence of this bitterness cannot be explained by tastants-mediated T2R signals in Type II taste cells. Intriguingly, we first demonstrated that the off-response of the PKD2L1 channel endogenously expressed in Type III taste cells to quinine may contribute to the long-lasting bitter perception. These results are important for a full understanding of the bitter taste perception. On the other hand, the bitter taste perception is associated with the viewpoint of drug discovery. The understanding of the bitterness response may lead to the development of medical drugs that reduce bitterness.

In conclusion, we suggest that the PKD2L1 channel in Type III taste cells is involved in the aftertaste perception of quinine. To our knowledge, this is the first report clarifying the ion channel contributing to a bitter aftertaste.

## Methods

### Chemicals

Quinine hydrochloride and denatonium benzoate were obtained from Fujifilm Wako Pure Chemical (Osaka, Japan) and dissolved in a buffer or normal water. Collagenase A and dispase II were from Roche (Basel, Switzerland). Elastase was from Worthington Biochemical (Lakewood, NJ, USA). DNase I was from Merck (Darmstadt, Germany). Lipofectamine 2000 was from Thermo Fisher Scientific (Waltham, MA, USA). Fura-2 acetoxymethyl ester, ethylene glycol-bis(2-aminoethyl ester)-N,N,N’,N’-tetraacetic acid (EGTA), and 4-(2-hydroxyethyl)-1-piperazineethanesulfonic acid (HEPES) were from Dojindo Laboratories (Kumamoto, Japan). Poly-L-lysine was from Sigma-Aldrich. All other reagents were of molecular biology grade or the highest grade of purity available.

### Cell culture and transfection

Human embryonic kidney HEK293T cells (kindly provided by Prof. Makoto Tominaga) were grown in Dulbecco's Modified Eagle's Medium (D-MEM with low glucose: Fujifilm Wako Pure Chemical) supplemented with 10% fetal bovine serum, 100 unit/ml penicillin, and 100 µg/ml streptomycin at 37 °C in a humidity-controlled incubator with 5% CO_2_.

The pCINeo/IRES-GFP expression vector containing mouse PKD2L1^24^ was transiently transfected into HEK293T cells using Lipofectamine 2000 according to the manufacturer’s instruction. Transfected HEK293T cells were detached from the plastic substrate and cultured on round coverslips (Matsunami Glass, Osaka, Japan) before electrophysiological experiments. The experiments were carried out with GFP-positive cells more than 36 h after the transfection.

### Electrophysiological experiments

Whole-cell patch-clamp recordings were performed using an Axopatch 200B patch-clamp amplifier (Molecular Devices, San Jose, CA, USA) connected with a Digidata 1550A digitizer (Molecular Devices). Clampex 10.6 software (Molecular Devices) was used for command pulse control and data acquisition. Currents were filtered at 1 kHz and digitized at 10 kHz. The data were analyzed with Clampfit 10.6 (Molecular Devices), Origin (OriginLab, Northampton, MA, USA), and WinASCD software (kindly provided by Dr. Guy Droogmans). Patch electrodes fabricated using a Flaming/Brown micropipette puller (P-97: Sutter Instrument, Novato, CA, USA) had a resistance of 2–3 MΩ when filled with the pipette solution. The access resistance was electrically compensated by 70% to minimize voltage errors.

As previously reported^17–19^, single-channel PKD2L1 currents were observed at negative potentials in the whole-cell recordings. After making whole-cell configurations, step pulses from − 100 to + 160 mV in 20-mV increments with a post-potential of − 100 mV were applied every 2 min. When the tail currents on repolarization to − 100 mV became stable, we started to measure the PKD2L1 currents at a holding potential of − 60 mV. The all-points amplitude histogram made from each 30-s recording was fitted with Gaussian functions to calculate the single-channel current amplitude. The channel activity (NPo, where N is the number of channels and Po is the open probability) was analyzed by dividing the average current amplitude for 30 s by the corresponding single-channel current amplitude.

The intracellular pipette solution consisted of 130 mM CsOH, 130 mM L-aspartate, 2 mM Na_2_ATP, 10 mM MgCl_2_·6H_2_O, 1 mM EGTA, and 10 mM HEPES (pH 7.3 adjusted with CsOH). The extracellular bathing solution contained 130 mM NaCl, 1 mM MgCl_2_·6H_2_O, 10 mM HEPES, and 40 mM D(−)-mannitol (pH 7.4 adjusted with NaOH).

### Animals

The GAD67-GFP knock-in (wild-type) and the PKD2L1-knockout with GAD67-GFP knock-in (PKD2L1-knockout) mice (kindly provided by Prof. Makoto Tominaga) were generated as previously described^14,16^. Adult male mice from 8 to 20 weeks old were used in the present experiments. All animal experiments were approved by the Animal Experiment Committee at the University of Toyama (A2013PHA-8, A2016PHA-11, A2019PHA-1, A2022PHA-1) and conducted in compliance with the Guidelines for the Care and Use of Laboratory Animals at the University of Toyama and the ARRIVE (Animal Research: Reporting of In Vivo Experiments) guideline.

### Isolation of taste cells

Mice were euthanized by cervical dislocation after intraperitoneal administration of 100 mg/kg sodium pentobarbital just before the experiments. All efforts were made to minimize suffering. The tongue cut from the euthanized mice was put in Tyrode’s solution composed of 135 mM NaCl, 5 mM KCl, 2 mM CaCl_2_·2H_2_O, 1 mM MgCl_2_·6H_2_O, 5 mM NaHCO_3_, 10 mM HEPES, 10 mM D(+)-glucose, and 10 mM Na pyruvate (pH 7.3 adjusted with NaOH). The Tyrode’s (Tyrode-enzyme) solution supplemented with 1 mg/ml collagenase A, 2.5 mg/ml dispase II, and 0.5 mg/ml elastase was injected under lingual epithelium containing circumvallate papillae. After the tongue was incubated in the Ca^2+^, Mg^2+^-free Tyrode’s (CMF-Tyrode) solution containing 2 mM EGTA instead of CaCl_2_·2H_2_O and MgCl_2_·6H_2_O for 20 min at room temperature, the lingual epithelium was exfoliated. The peeled epithelium was treated with the Tyrode-enzyme solution for 3 min, the CMF-Tyrode solution for 5 min, and the Tyrode’s (Tyrode-DNase) solution with 0.25 mg/ml DNase I for 2 min. The epithelium was pinned upside down in a sylgard-coated dish filled with Tyrode’s solution. Taste buds were collected using a fire-polished micropipette (tip diameter: 80 µm) with mild suction. The taste buds were digested with 0.25% trypsin–EDTA at 37 °C for 20 min and incubated in the Tyrode-DNase solution for 1 min. Then, taste cells were isolated from the taste buds by alternately applying positive pressure and negative pressure using another fire-polished micropipette (tip diameter: 30 µm) on a glass-based dish coated with poly-L-lysine.

### Ca^2+^-imaging analysis

Isolated taste cells were incubated in Tyrode’s solution containing 5 µM Fura-2 acetoxymethyl ester at 37 °C for 30 min. Ca^2+^ imaging was carried out using the AQUACOSMOS/RATIO system (Hamamatsu Photonics, Hamamatsu, JAPAN). The Fura-2 fluorescence ratio excited at 340 and 380 nm was measured. Since Type III taste cells express voltage-dependent Ca^2+^ channels^1^, Tyrode’s solution (high-K^+^) with a high concentration of K^+^ was used to identify Type III taste cells. The high-K^+^ Tyrode’s solution contained 90 mM NaCl, 50 mM KCl, 2 mM CaCl_2_·2H_2_O, 1 mM MgCl_2_·6H_2_O, 5 mM NaHCO_3_, 10 mM HEPES, 10 mM D(+)-glucose, and 10 mM Na pyruvate (pH 7.3 adjusted with NaOH).

### Quantitative polymerase chain reaction (qPCR) analysis

Total RNA was extracted from the lingual epithelium containing circumvallate papillae of the wild-type and the PKD2L1-knockout mice with SV Total RNA Isolation System (Promega, Madison, WI, USA) and transcribed into cDNA using Random Primer (9mer) and ReverTra Ace (TOYOBO, Osaka, Japan). Quantitative PCR experiments were performed by Luna Universal qPCR Master Mix (New England Biolabs, Ipswich, MA, USA) in a real-time PCR system (Mx3000P: Agilent Technologies, Santa Clara, CA, USA). The following thermal conditions were used: an initial denaturation of 95 °C for 60 s and the next 50 cycles of 95 °C for 15 s and 60 °C for 30 s. The designed primer pairs were as follows: for mouse β2-adrenergic receptor: 5’- atagcaacggcagaacggac-3’ and 5’-cacaaagccttccatgcctg-3’ and for glyceraldehyde-3-phosphate dehydrogenase (GAPDH): 5’-aacctgccaaatatgatgac-3’ and 5’-ataccaggaaatgagcttga-3’.

### Intracellular cAMP measurements

The isolated lingual epithelium containing circumvallate papillae from the wild-type and the PKD2L1-knockout mice were incubated in Tyrode’s solution with 30 µM quinine for 3 min. The samples were treated with 0.1 M HCl for 20 min and homogenated. The homogenates were centrifuged at 1000 X g for 10 min and the supernatants were used for cAMP EIA Kit (Cayman, Ann Arbor, MI, USA). All the samples were acetylated to detect a lower concentration of cAMP according to the instructions.

### Two-bottle preference tests

Before the two-bottle preference experiments, mice were trained to access two bottles of normal water equally. In the long-term experiments for 48 h, mice were presented with normal water and water containing a bitterant such as quinine or denatonium. After 24 h, the position of the bottles was manually reversed. The amount of intake in each bottle was measured daily. A ratio of tastant intake to the total intake was indicated as a preference ratio. In the short-term experiments, mice deprived of water for 2 h were initially given access to two bottles of bitterant-containing water for 15 min and then manually replaced with a bottle of normal water and a bottle of bitterant-containing water for 15 min. The amount of intake in each bottle was measured.

### Lick tests

Before the lick tests, mice were deprived of normal water for 23 h. In the experiments, mice were three times manually presented with a bottle of test solution for 10 s. To adapt mice to this protocol, the lick tests with normal water were carried out for 3–5 days. In the practiced mice, bitterant-containing water and normal water were given alternately. The counts and duration of licks were measured in a self-made lick analysis system^27^.

### Statistics

Data are presented as means ± S.E.M. of n observations. The normality of the data was confirmed by the Kolmogorov–Smirnov test. Statistical differences in the data were evaluated by Wilcoxon signed-rank test, paired or unpaired Student’s t-test, and one-way ANOVA with Tukey’s post-hoc analysis. The differences were considered significant at *p* < 0.05.

## Data Availability

All data generated or analyzed during this study are included in this published article.
